# Morphological Predictive Features on Spectral-Domain Optical Coherence Tomography for Visual Outcomes in Neovascular Age-Related Macular Degeneration Treated with Ranibizumab

**DOI:** 10.1155/2018/7438083

**Published:** 2018-06-26

**Authors:** Georges Azar, Benjamin Wolff, Flore De Bats, Jeremie Halfon, Mate Streho, Sarah Tick, Laurent Castelnovo, Guillaume Michel, Helene Masse, Vivien Vasseur, Marwan Sahyoun, Martine Mauget-Faÿsse

**Affiliations:** ^1^Eye & Ear Hospital International, Beirut, Lebanon; ^2^Holy Spirit University of Kaslik (USEK), Faculty of Medicine, Kaslik, Lebanon; ^3^Saint Joseph University (USJ), Faculty of Medicine, Beirut, Lebanon; ^4^Rothschild Ophthalmological Foundation, 25 rue Manin, 75940 Paris Cedex 19, France; ^5^Pole Vision Center, Clinique du Val-d'Ouest, 39 chemin de la Vernique, 69130 Écully, France; ^6^Halles de Tours Ophthalmological Center, 13 place Gaston-Paillhou, 37000 Tours, France; ^7^Explore Vision Center, 2 rue Grandes-Terres, 92500 Rueil-Malmaison, France; ^8^Quinze-Vingts Ophthalmology National Center, 28 rue de Charenton, 75571 Paris Cedex, France; ^9^Maison Rouge Ophthalmological Center, 6 rue de l'Eglise, 67000 Strasbourg, France; ^10^Nantes University Hospital Center, 8 quai Moncousu, 44000 Nantes, France

## Abstract

**Purpose:**

To identify spectral-domain optical coherence tomography (SD-OCT) predictive morphological features for the outcome of Ranibizumab therapy for neovascular age-related macular degeneration (AMD).

**Methods:**

This is a retrospective multicentric study that involved 64 eyes with naïve AMD. Patients who received three monthly intravitreal injections of Ranibizumab were stratified into (1) “responders” [≥ 5 letters gain on Early Treatment Diabetic Retinopathy Study (ETDRS) scale] and (2) “nonresponders” (< 5 letters gain). Best-corrected visual acuity (BCVA) and SD-OCT morphological features were compared at baseline and one month after three consecutive injections of Ranibizumab. Univariate and multivariate analyses were carried out to correlate these morphological features with the change in BCVA.

**Results:**

Among the 64 patients enrolled, 40 (62.5%) were “responders” and 24 (37.5%) “nonresponders”. Age, sex, and BCVA were comparable between both groups. A multivariate correlational analysis found that subfoveal choroidal thickness (SFCT) and the presence of pigment epithelial detachment (PED) > 250 *μ*m at baseline were two independent prognostic indicators of final BCVA. No other SD-OCT morphological studied features seem to affect final BCVA after Ranibizumab treatment.

**Conclusion:**

SFCT and the presence of PED > 250 *μ*m are two significant biomarkers that may predict improvement after Ranibizumab therapy for AMD. These markers may guide ophthalmologists' treatment decision under financial constraints and limited time.

## 1. Introduction

Exudative age-related macular degeneration (AMD) is a leading cause of irreversible loss of central vision in developed countries [1–3]. By 2040, around 288 million adults worldwide are expected to develop AMD [4]. Therapy of exudative AMD with antivascular endothelial growth factor (VEGF) intravitreal injections, which include Bevacizumab (Avastin; Genentech, San Francisco, CA, USA), Ranibizumab (Lucentis, Novartis International AG, Basel, Switzerland), and Aflibercept (Eylea; Regeneron, Tarrytown, NY, USA) has evolved to a widespread and effective treatment in the last years, allowing prevention of vision loss and the possibility for vision improvement with regular use of those agents over 2 years [5–8].

In an effort to avoid potential complications and optimize outcomes, different regimens have been investigated [9–16]. Nevertheless, despite those established regimens that have been found to improve visual acuity (VA) in up to one-third of patients with subfoveal neovascular AMD [9–11], 10% to 15% of patients continue to lose vision and more than half of patients will demonstrate a gradual loss after an initial gain [3, 9, 11, 17, 18]. Therefore, identifying predictive factors of good and bad responders with anti-VEGF treatment allows for more accurate prediction of prognosis based on patients' baseline and imaging characteristics.

Recent reports have shown that the final visual outcomes among treated patients depend on several baseline factors. Some of those reported factors are age, smoking [19, 20], genetic factors such as complement factor H (CFH) and the age-related macular susceptibility 2 (ARMS2) variants Y402H and A69S [21, 22], baseline visual acuity [23], and neovascular lesion baseline characteristics such as large choroidal neovascular (CNV) area or CNV subtype (classic versus occult, predominantly versus minimally classic lesion subtypes).

With the advent of higher resolution in retinal imaging, better axial resolution, faster acquisition time, and real-time averaging of scanning laser ophthalmoscopic images, current improvement in spectral-domain optical coherence tomography (SD-OCT) devices has led to a better assessment of the retinal microstructures, and surrogative OCT biomarkers for the efficacy of certain therapy have become more and more feasible [24, 25].

A few publications have already focused on identifying some baseline OCT characteristics such as total foveal thickness, subretinal fluid (SRF) thickness, retinal pigment epithelial (RPE) elevation, the presence of retinal angiomatous proliferation (RAP), and the presence of geographic atrophy (GA) that can predict response to anti-VEGF therapy, specifically Ranibizumab [26–31]. However, finding precise, measurable, and reproducible OCT morphological parameters that could allow physicians to better anticipate the clinical outcome of patients treated by Ranibizumab injections starting the time of diagnosis is crucial and yet to be determined.

The main purpose of this report was to further explore and study baseline measurable phenotypic criteria on SD-OCT, which may serve as predictors for good and poor visual outcomes, in patients with exudative AMD receiving three loading doses of intravitreal Ranibizumab injections.

## 2. Methods

### 2.1. Study Design and Participants

This is a retrospective, observational, multicentric study that involved 64 patients treated for naïve subfoveal exudative AMD, who received three monthly intravitreal injections with Ranibizumab at the Maison Rouge Ophthalmological center, Strasbourg, France, Pole Vision Center, Ecully, France, Tours Ophthalmological Center, Tours, France, Quinze-Vingts National Ophthalmology Hospital, Paris, France, Explore Vision Center, Rueil-Malmaison, France, and the Nantes University Hospital Center, Nantes, France, between January 2016 and January 2017. Approval was obtained by the Research and Development Department, the Institutional Review Board, and the Ethics Committee at each participating study site, as a retrospective study not requiring informed consent. The study adhered to the tenets of the Declaration of Helsinki and the International Conference of Harmonization Good Clinical Practice guidelines. Key inclusion criteria included age older than 60 years; patients with naïve exudative AMD who had evidence of subfoveal or juxtafoveal choroidal neovascularization (CNV) lesions affecting the fovea and who had received 3 consecutive injections with Ranibizumab; and a minimum follow-up time of 6 months. Key exclusion criteria included age younger than 60 years; high myopia (defined as spherical equivalent > 6.00 diopters or axial length > 26.5 mm); the presence of uncontrolled glaucoma; the presence of significant epiretinal membrane, vitreomacular traction, and polypoidal choroidal vasculopathy (PCV) in the study eye on baseline OCT imaging; the presence of any other ocular comorbidities such as retinal artery or vein occlusions, uveitis, and macular edema from any other cause; and ocular surgery during the follow-up period of treatment.

### 2.2. Data Collection

The medical charts of all eligible patients were reviewed. Demographic information was collected for each patient, including age at first injection; the presence of systemic comorbidities such as diabetes mellitus or systemic hypertension; and history of glaucoma. Data concerning clinical examinations at baseline and then monthly after three consecutive injections of Ranibizumab were also collected. This included measurement of best-corrected visual acuity (BCVA), slit-lamp examination, direct and indirect ophthalmoscopy, and Goldmann applanation tonometry. Visual acuity was measured using the Early Treatment Diabetic Retinopathy Study- (ETDRS-) like charts at an initial testing distance 4 m, done by one experienced tester after standardized refraction. Moreover, procedure analyses from color fundus photograph images, fundus fluorescein angiography (FFA), and morphologic analysis with SD-OCT were also recorded.

### 2.3. Morphologic Analysis with Optical Coherence Tomography

**Figure 1:**
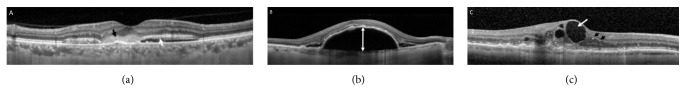
Spectral-domain optical coherence tomography (SD-OCT) B-scan images (Heidelberg Engineering GmBH, Heidelberg, Germany) of patients with exudative age-related macular degeneration (AMD) showing subretinal hyperreflective exudation ((a) black arrow), subretinal fluid ((a) white arrow), maximal pigment epithelial detachment's height as measured from the top of the retinal pigment epithelium to the Bruch membrane (b), intraretinal fluid ((c) white arrow), and hyperreflective dots ((c) black arrows).

Spectral-domain OCT imaging was performed through a dilated pupil using a Heidelberg Spectralis OCT machine (Heidelberg Engineering GmBH, Heidelberg, Germany) with the established “posterior pole” protocol, which provides high-speed scans with a dimension of 30° x 25° and a 120 *μ*m B-scan spacing. Each line scan had a resolution of 512 A-scans per section, and automated real-time tracking was turned on throughout the scan acquisition. The maximum width of each of the following characteristics was measured using the caliper scale provided by the software of the Spectralis OCT machine (Figure 1): central retinal thickness (CRT), intraretinal fluid (IRF), subretinal fluid (SRF), pigment epithelial detachment (PED), subretinal hyperreflective exudation (SHE), and subfoveal choroidal thickness (SFCT). Other characteristics specifically noted during each visit were the presence or absence of ellipsoid zone (EZ) presumed to represent the inner segment of photoreceptors in the foveal area, areas of absence of retinal pigment epithelial (RPE), hyperreflective dots in the retinal layers, cystoid macular edema (CME), retinal atrophy, and the type of CNV. The IRF was defined as the presence of an optically empty area within the retina, whereas the SRF was defined as an optically empty area directly internal to the RPE/Bruch membrane complex but external to the outer retina. PED was identified as an elevation of the RPE overlying a hyporeflective (optically empty) or iso- or hyperreflective area, and maximal PED measurement was done from the top of the RPE to the Bruch membrane [27]. SHE is a novel type of subretinal hyperreflective material located in the subretinal space. It likely represents a sign of active neovascular AMD [32]. The EZ was defined as the second hyperreflective (HR) layer above the hyperreflective band of the RPE [33]. Hyperreflective dots are small particles scattered throughout the retinal layers, more frequently in the outer retina [34]. Finally, the classification of CNV, including type 1 (sub-RPE), type 2 (subretinal), type 3 (intraretinal), and type 4 (mixed), was made independently by two experienced retina specialists (BW and FDB) who evaluated the presenting color photographs, FA, and SD-OCT. Each neovascular lesion was classified according to the FA alone and with the anatomic classification system as previously detailed by Jung and associates [35]. All the above morphological features were evaluated at baseline and one month after the 3 loading doses of Ranibizumab during the 3-month visit after commencement of Ranibizumab treatment.

### 2.4. Predictive Value of OCT Morphological Features

To further explore and study baseline measurable phenotypic criteria on SD-OCT, which may serve as potential predictors for good and poor visual outcomes, specifically after the initial 3 loading dose injections of Ranibizumab, patients were further stratified into two groups depending on the early initial response of BCVA: (1) patients who gained 5 or more than 5 letters on ETDRS scale (“responders”) and (2) patients who gained less than 5 letters on ETDRS scale (“nonresponders”). A multivariate analysis using SD-OCT morphological variables at baseline (D0) and at 3 months (M3) was then done between the two groups in order to assess the potential positive predictors of good initial response to Ranibizumab.

### 2.5. Ranibizumab Injection Procedure

All patients diagnosed with AMD received three initial consecutive monthly intravitreal injections of Ranibizumab (0.5 mg/0.05 mL) under aseptic conditions. Prior to injection, all eyes were given topical proparacaine hydrochloride (0.5%) and topical 5% povidone-iodine solution. Injections were performed 3.5 mm to 4.0 mm posterior to the limbus with a 30-gauge needle through the pars plana.

### 2.6. Statistical Analysis

Subjects who were ineligible for the clinical study based on the inclusion and exclusion criteria were excluded, leaving a total of 64 patients available for data analysis. As previously described, two groups were formed on the basis of BCVA change after 3 loading doses of Ranibizumab treatment: (1) “responders” and (2) “nonresponders”. The comparison of baseline characteristics, visual outcomes, and morphologic OCT outcomes was performed using analysis of variance for most continuous measures and Monte Carlo exact tests for categorical measures. Multiple linear regression was performed on the variables taken into account on univariate analysis to detect independent prognostic indicators, after which a parsimonious model of VA was performed using the significant predictors. The reliability of the grading of each OCT feature was assessed using weighted *κ* statistics and intraclass correlation coefficients. *κ* statistics were interpreted using ranges suggested by Landis and Koch: 0 to 0.20, slight agreement; 0.21 to 0.40, fair agreement; 0.41 to 0.60, moderate agreement; 0.61 to 0.80, substantial agreement; and more than 0.80, almost perfect agreement [36]. The intraclass correlation coefficient was considered reliable if the values were between 0.4 and 0.75, and values over 0.75 were considered excellent. All analyses were conducted using SAS statistical software, version 9.4 (SAS Institute Inc., Cary, NC). A 2-sided P value <0.05 was considered to be statistically significant.

## 3. Results

### 3.1. Baseline Characteristics

Data from 64 eyes from participants who were newly diagnosed with treatment-naïve neovascular AMD in at least one eye and treated with Ranibizumab between January 2016 and January 2017 were included in the analysis. For the whole group at the baseline visit, before the start of the treatment, the mean age was 79.1 ± 1.7 years, with 43 (67.2%) women and 21 (32.8%) men. The CNV subtypes recorded on fluorescein angiography at presentation included 36 cases of type 1 CNV (sub-RPE) (56.3%), 14 cases of type 2 CNV (subretinal) (21.9%), 7 cases of type 3 CNV (intraretinal) (10.9%), and 7 cases of type 4 CNV (mixed) (10.9%). Thirty-four of the 64 eyes (53.1%) were pseudophakic. These baseline characteristics are further detailed in Table 1 according to patients' stratification into “responders” and “nonresponders”. All patients' data were evaluated the day before the first Ranibizumab injection (D0) and one month after three initial consecutive monthly intravitreal Ranibizumab injections (M3).

### 3.2. Overall Response and Time Course of Individual Morphological Parameters

**Figure 2:**
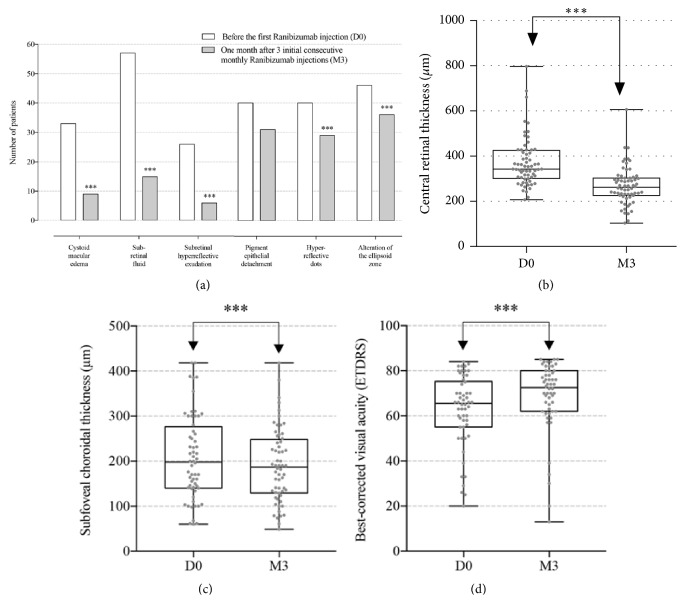
Variation of morphologic spectral-domain optical coherence tomography (SD-OCT) parameters documented in a qualitative manner (a) for the presence of cystoid macular edema, subretinal fluid, subretinal hyperreflective exudation, pigment epithelial detachment, hyperreflective dots and alteration of the ellipsoid zone, and quantitative manner for central retinal thickness [measured in *μ*m (b)], subfoveal choroidal thickness [measured in *μ*m (c)], and best-corrected visual acuity (BCVA) [using the Early Treatment Diabetic Retinopathy Study (ETDRS) chart (d)], before the first Ranibizumab injection (D0) and one month after 3 initial consecutive monthly Ranibizumab injections (M3). All parameters were found to change significantly (marked as *∗∗∗*) after Ranibizumab injections except for pigment epithelial detachment.

Figure 2 summarizes overall baseline clinical and morphological characteristics of patients analyzed at baseline (D0) and 3-month follow-up (M3). The mean VA at D0 was 61.6 ± 10.4 and that at M3 was 67.4 ± 8.2 ETDRS letters (p<0.001). The mean CRT was 375.37 ± 12 *μ*m at D0 and 270.7 ± 22 *μ*m at M3 (p<0.001), whereas the SFCT was 203.7 ± 12 *μ*m at D0 and 193.6 ± 13 *μ*m at M3 (p=0.05). Among the morphological features on SD-OCT, the presence of CME [33 cases (51.6%) at D0 and 9 cases (14.1%) at M3, p<0.001], SRF [57 cases (89.1%) at D0 and 15 cases (23.4%) at M3, p<0.001)], SHE [(26 cases (40.6%) at D0 and 6 cases (9.4%) at M3, p<0.001)], and hyperreflective dots [40 cases (62.5%) at D0 and 29 cases (45.3%) at M3, p<0.05)] and absence of intact EZ [46 cases (71.9%) at D0 and 36 cases (56.3%) at M3, p<0.05)] were found to significantly change between the day of presentation and 1 month after the third Ranibizumab injection. As also shown on Figure 2, the presence of PED [40 cases (62.5%) at D0 and 31 cases (48.4%) at M3, p= 0.11)] did not vary significantly at D0 and M3 after Ranibizumab injections.

According to their early response after the initial 3 loading dose injections of Ranibizumab, 40 patients (62.5%) were found to be “responders” and gained 5 or more than 5 letters on ETDRS scale, and 24 patients (37.5%) were found to be “nonresponders” and gained less than 5 letters on ETDRS scale.

At the moment of presentation, BCVA was 58 ± 8.0 ETDRS letters for the “responders” group and 61 ± 7.2 ETDRS letters for the “nonresponders” group (p=0.27).

By stratifying our patients into those two groups, the CNV subtypes recorded on fluorescein angiography at presentation included type 1 CNV (sub-RPE) in 20 cases (50%) in the “responders” group and 16 cases (66.7%) in the “nonresponders” group, type 2 CNV (subretinal) in 10 cases (25%) in the “responders” group and 4 cases (16.7%) in the “nonresponders” group, type 3 CNV (intraretinal) in 5 cases (12.5%) in the “responders” group and 2 cases (8.3%) in the “nonresponders” group, and finally type 4 CNV (mixed) in 5 cases (12.5%) in the “responders” group and 2 cases (8.3%) in the “nonresponders” group (Table 1).

### 3.3. Predictive Value of OCT Morphological Features

**Figure 3:**
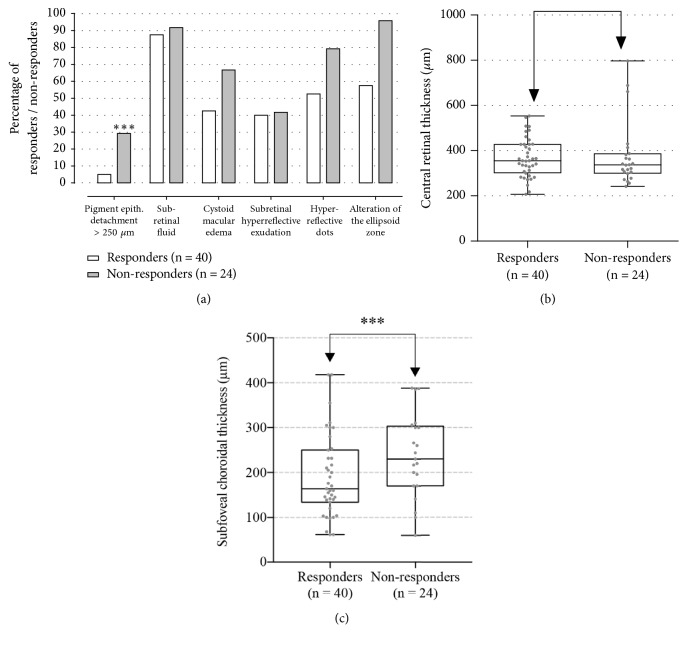
The results of the multivariate correlational analysis of spectral-domain optical coherence tomography (SD-OCT) qualitative morphological features ((a) presence of pigment epithelial detachment (PED) > 250 *μ*m, subretinal fluid, cystoid macular edema, subretinal hyperreflective exudation, hyperreflective dots, and alteration of the ellipsoid zone) and quantitative morphological features [(b) central retinal thickness (measured in *μ*m) and (c) subfoveal choroidal thickness (SFCT, measured in *μ*m)] between both “responders” and “nonresponders” groups. The presence of PED > 250 *μ*m and SFCT was found to statistically affect the response to Ranibizumab injections (marked as *∗∗∗*) in age-related macular degeneration patients (p-value was derived from analysis of variance for continuous variables and Monte Carlo exact tests for categorical analysis).

The results of the multivariate correlational analysis of SD-OCT morphological features between the two groups are depicted in Figure 3. Briefly, the presence of a high protruding PED (>250 *μ*m) at the moment of presentation was found to be statistically significant between “responders” and “nonresponders” [2 cases (5%) and 7 cases (29.2%), respectively, p<0.001)], meaning that when a baseline PED >250 *μ*m is present, the initial response to 3 loading doses of Ranibizumab is expected to be poor. Another morphological feature that was significantly correlated with a better BCVA response was a SFCT that was significantly lower in the “responders” group compared to the “nonresponders” group (191.6 ± 91.3 *μ*m and 236.2 ± 93.5 *μ*m, respectively, p=0.039). When analyzing the other OCT morphological features, the presence of SRF [35 cases (87.5%) in the “responders” group, 22 cases (91.7%) in the “nonresponders” group, p=0.61)], CME [17 cases (42.5%) in the “responders” group, 16 cases (66.7%) in the “nonresponders” group, p=0.06)], SHE [16 cases (40.0%) in the “responders” group, 10 cases (41.7%) in the “nonresponders” group, p=0.63)], and hyperreflective dots [21 cases (52.5%) in the “responders” group, 19 cases (79.2%) in the “nonresponders” group, p=0.51)] and the presence of EZ alterations [23 cases (57.5%) in the “responders” group, 23 cases (95.8%) in the “nonresponders” group, p=0.12)] were not found to be statistically significant between the 2 groups. Moreover, the mean of CRT was not statistically different between both groups (368.7 ± 87.2 *μ*m in the “responders” group and 376.0 ± 141.1 *μ*m in the “nonresponders” group, respectively, p=0.80).

## 4. Discussion

Identification of reliable and predictive morphological factors may enable physicians to council patients with AMD more efficiently concerning probability of improvement of any anti-VEGF therapy. Currently, due to advances in the resolution of OCT devices, in vivo imaging and subtle changes of retinal layers become more and more feasible in everyday practice. However, analyses of the correlations between baseline OCT characteristics and visual outcomes in eyes treated with Ranibizumab for exudative AMD remain limited. In this study, we report the baseline morphological factors that predict either good or poor VA after Ranibizumab therapy for treatment-naïve exudative AMD using an ETDRS scale. Previous studies have retrospectively analyzed some of those factors but provided conflicting results.

We studied possible anatomical markers such as the presence of CME, SRF, and CRT, which show conflicting results in the literature. While Gamulescu* et al. *showed that CME was associated with a poorer visual prognosis as well as lower grades of continuity of outer layers than SRF [26], none of those two factors seem to significantly affect the visual outcome in our series. Furthermore, three previous studies also failed to detect significant correlation between the presence of SRF and visual outcomes [28, 37, 38]. However, while Segal* et al. *found a significant and positive correlation between SRF and visual outcomes [3], they found no significant correlation regarding CME and CRT.

While previous reports have indicated that the integrity of the photoreceptor layer represented by the EZ is a good predictor of final VA in eyes with AMD treated with anti-VEGF [27, 39–41], our results did not find any correlation between these two parameters. Furthermore, we did not find that SHE was a surrogate marker of poor VA. Similar findings to those mentioned above were also obtained by Segal* et al. *[3], Mathew* et al.* [27], and Shah* et al*. [32]. Moreover, the presence of hyperreflective dots in our series did not seem to contribute significantly to VA. Although they were more present in “nonresponders” eyes, along areas of fluid accumulation, their presence did not significantly correlate with VA in our study.

Of note, when examining the CNV type effect as evaluated on FA (sub-RPE versus subretinal versus intraretinal versus mixed), we did not find any significant correlation between the different types of CNV and visual outcomes after Ranibizumab injections. This goes against the results of Chae* et al.* who found that eyes having type 1 CNV at baseline were more likely to maintain good vision after anti-VEGF therapy [23].

Interestingly, in this current study, we found a significant and negative correlation between the presence of high protruding PED (> 250 *μ*m) and the number of letters gained on ETDRS scale after three injections of Ranibizumab. Rather than evaluating the presence or absence of PED, we speculate that the height and area of PED may serve as a better predictor of potential improvement of BCVA following Ranibizumab injections. To date, there have been no reports in the literature on the role of high protruding PED on SD-OCT as a surrogate marker of poor VA.

On the other hand, we interestingly found that mean SFCT at presentation was significantly higher in the “nonresponders” group compared to the “responders” group, suggesting that SFCT may be a surrogate marker of poor VA after Ranibizumab treatment in AMD patients. To the best of our knowledge, this is the first report to focus on those two OCT morphological factors, which have never been previously described as potential prognostic parameters.

Given the differences in baseline BCVA between “responders” and “nonresponders”, some differences in visual response related to studied morphological parameters could have been attributable to a ceiling effect. That is, owing to their better baseline VA, the “nonresponders” group may have less capacity for improvement than “responders” group, therefore achieving smaller gains. However, this presumed ceiling effect was not statistically found to be significant between group, suggesting therefore that it is unlikely that the baseline BCVA differences are sufficient to explain the differential responses between both groups.

This series is limited by both its retrospective nature and by its relative small sample size. Therefore, prospective evaluation is required on a larger scale to determine more precise, measurable, and reproducible anatomical and morphological factors on SD-OCT, ideally in multicenter studies. Ultimately, a randomized controlled trial comparing groups of patients with different morphological factors, who have the same baseline characteristics, and receiving a unified anti-VEGF protocol for AMD will provide more validated data on the role of the morphological factors on SD-OCT as predictors for good and poor visual outcomes after anti-VEGF therapy.

In conclusion, this report suggests the role of morphological quantitative and qualitative OCT parameters with respect to visual prognosis after Ranibizumab therapy for exudative AMD. Specifically, we identified the presence of a high protruding PED (> 250 *μ*m) and SFCT as significant markers predicting improvement in VA. Final BCVA can therefore be predicted based on a model that incorporates the presence or absence of a high protruding PED and SFCT. These prognostic indicators may be incorporated in retreatment criteria to define better need for further injections. It also suggests the possibility of guidance in future patient-individualized disease management of any antiangiogenic strategy to optimize functional outcomes and limit socioeconomic burden.

## Figures and Tables

**Table 1:** Patient demographic and baseline characteristics.

	**Group**		*P-value*
	**“Responders”**	**“Nonresponders”**	
	**(n = 40)**	**(n = 24)**	
Age at first injection, years (mean ± SD)	81.3 ± 2.6	76.2 ± 4.2	0.30
Sex, n (%)			
*Male*	15 (37.5)	6 (25.0)	0.33
*Female*	25 (62.5)	18 (75.0)	0.29
Best-corrected visual acuity, ETDRS (mean ± SD)	58 ± 8.0	61 ± 7.2	0.27
Central retinal thickness, *μ*m (mean ± SD)	368.7 ± 87.2	376.0 ± 141.1	0.80
CNV type on FA, n (%)			
*Type 1 CNV*	20 (50.0)	16 (66.7)	0.37
*Type 2 CNV*	10 (25.0)	4 (16.7)	0.28
*Type 3 CNV*	5 (12.5)	2 (8.3)	0.42
*Type 4 CNV*	5 (12.5)	2 (8.3)	0.42

n, number; SD, standard deviation; y, years; ETDRS, Early Treatment Diabetic Retinopathy Study; CNV, choroidal neovascularization; FA, fluorescein angiography; Type 1 CNV, sub-RPE (retinal pigment epithelium) CNV; Type 2, subretinal CNV; Type 3, intraretinal CNV; Type 4, Mixed CNV.

## Data Availability

The data used to support the findings of this study are available from the corresponding author upon request.

## Additional Points

*Summary Statement. *The baseline morphological criteria on spectral-domain optical coherence tomography, which may serve as predictors for outcomes in patients with exudative age-related macular degeneration receiving Ranibizumab injections, are studied. The presence of a high protruding pigment epithelial detachment and subfoveal choroidal thickness were found to be significant markers predicting improvement in visual acuity.
